# Comparison of children’s physical activity profiles before and after COVID-19 lockdowns: A latent profile analysis

**DOI:** 10.1371/journal.pone.0289344

**Published:** 2023-11-27

**Authors:** Ruth Salway, Frank de Vocht, Lydia Emm-Collison, Kate Sansum, Danielle House, Robert Walker, Katie Breheny, Joanna G. Williams, William Hollingworth, Russell Jago

**Affiliations:** 1 Centre for Exercise, Nutrition & Health Sciences, School for Policy Studies, University of Bristol, Bristol, United Kingdom; 2 Population Health Sciences, Bristol Medical School, University of Bristol, Bristol, United Kingdom; 3 The National Institute for Health Research, Applied Research Collaboration West (NIHR ARC West), University Hospitals Bristol and Weston NHS Foundation Trust, Bristol, United Kingdom; 4 Communities and Public Health, Bristol City Council, Bristol, United Kingdom; 5 NIHR Bristol Biomedical Research Centre, University Hospitals Bristol and Weston NHS Foundation Trust and University of Bristol, Bristol, United Kingdom; Yonsei University - Wonju Campus, REPUBLIC OF KOREA

## Abstract

Physical activity is important for children’s health, but moderate to vigorous physical activity (MVPA) declines with age. COVID-19 lockdowns resulted in reduced MVPA and increased sedentary time among children. Characterising children’s activity patterns may help identify groups who are most likely to be inactive post-lockdown. Data were combined from a pre-COVID-19 cohort study on children aged 5–6 years (Year1: n = 1299), 8–9 years (Year4: n = 1223) and 10–11 years (Year6: n = 1296) and cross-sectional post-lockdown data from a natural experiment on 10-11-year-olds in 2021 (Year6-W1: n = 393) and 2022 (Year6-W2: n = 436). The proportions of time spent in MVPA, light physical activity (LPA) and sedentary time on weekdays and weekends were derived from accelerometer data. Latent class analysis was used to identify activity profiles pre and post-lockdown, and estimate pre-COVID-19 transitions between Year4 and Year6. We identified six pre-COVID-19 activity profiles in Year6, including a new profile characterised by very low MVPA and high sedentary time (19% of children). There was substantial movement between profiles at Year4 and Year6, with 45% moving to a profile with lower MVPA. Likelihood ratio tests suggested differences in Year6 activity profiles pre and post-lockdown, with a new post-lockdown profile emerging characterised by higher LPA. The percentage of children in the least active profiles (where under 20% meet UK physical activity guidelines), rose post-lockdown, from 34% pre-COVID-19 to 50% in 2021 and 40% in 2022. We also saw gender and socioeconomic gaps widen, and increased separation between high and low physical activity levels. Children’s physical activity has changed post-COVID-19, in terms of who is being active and how. The impact varies by activity profile, which is influenced by gender and socio-economic position. A greater understanding of these differences and targeting of low active groups is needed to increase both individual and population levels of physical activity.

## Introduction

Increasing physical activity and decreasing sedentary time have both been found to be important for children’s health, with physical activity associated with emotional well-being, better cardiometabolic health and reduced risk of obesity [1, 2], while sedentary time is independently associated with increased adiposity [2, 3]. Moreover, low physical activity in childhood tracks into adulthood [4], leading to increased risk of heart disease, type 2 diabetes and some cancers [5, 6]. Despite this, children’s daily moderate to vigorous physical activity (MVPA) decreases throughout primary school by an average of 2 minutes per year. Over the same period daily sedentary time increases by an average of 13 min per year [7], and by age 11, the majority of children do not meet national UK guidelines of an average of 60 minutes of MVPA per day [7]. The COVID-19 pandemic has further impacted on children’s physical activity, with global reductions in physical activity and increases in sedentary time during periods of lockdown and restrictions [8–12]. In the UK, schools, leisure facilities and non-essential businesses were closed between 23^rd^ March and 13^th^ May 2020, with a second lockdown between 6^th^ January and 3^rd^ March 2021. After restrictions were lifted in 2021, MVPA initially remained lower and sedentary time higher compared to pre-pandemic [12–15]. In 2022, children’s sedentary time remains higher than pre-pandemic by 13 mins [16]. Qualitative data have suggested the emergence of a new normal for child physical activity, with a stronger emphasis on structured activity, such as active clubs, rather than unstructured activity, such as free play, and highlight the possibility of some groups who are at higher risk of being inactive [17, 18]. As still only 41% of 10-11-year olds children meet UK physical activity guidelines [16], there is an urgent need to increase children’s activity levels to improve health. A key component of this is understanding whether patterns of children’s physical activity have changed after the lockdowns and if so, how.

The associations between MVPA, light intensity physical activity (LPA) and sedentary behaviour are complex [19], and studying average levels of these behaviours in isolation may not give a complete picture of activity patterns [20]. For example, some children who engage in very high levels of MVPA also have high sedentary time. Latent class analysis [21] is a data-driven technique that has been used to characterise groups of children who share similar patterns of physical activity and sedentary time [22–25]. Identifying these activity profiles and exploring how they may change over time is important for designing behaviour change interventions as it facilitates the targeting of intervention approaches to those at greatest need.

Typical profiles include high physical activity/low sedentary time, high physical activity/high sedentary time and low physical activity/high sedentary time, with differences by gender and age [24, 25]. In a related study, we identified five activity profiles for children aged 5–6, and six profiles for the same children aged 8–9, ranging from very active to inactive [23], and with substantial movement between activity profiles between ages. We also saw strong gender differences in activity profile membership, with a higher proportion of boys in the more active profiles and girls in the more inactive profiles.

The aims of this paper are twofold. First, to extend our previous analyses to see if activity profiles change between ages 8–9 and age 10–11 pre-COVID-19. Second, to repeat the analysis in a post-lockdown sample to explore whether activity profiles at age 10–11 have changed post-pandemic, and if so in what way, either in terms of the profiles themselves or the proportions of children who fall into each. This will allow us characterise children’s activity patterns to identify groups who are most likely to be inactive post-lockdown, and thus at risk of poorer health and well-being. If physical activity patterns have changed, different strategies to increase children’s physical activity may be needed in future, and such groups may benefit from targeted strategies.

## Methods

**Fig 1:**
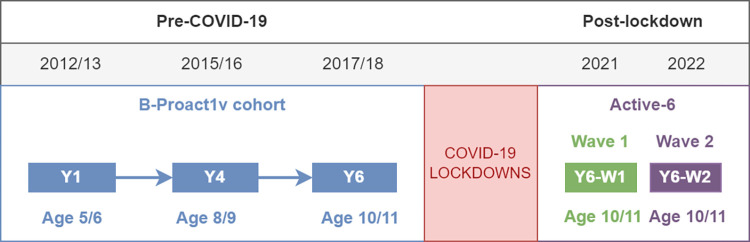
Timeline of data collection points.

This is an exploratory study, which combines data from two studies. B-Proact1v [7, 26, 27] was a longitudinal cohort study of physical activity and sedentary behaviours of children aged 5–11 years and their parents. Data were collected in three phases between January 2012 and May 2018, when the children were aged 5–6 years (in Year 1 of primary school in the UK; Y1), 8–9 years (Y4) and 10–11 years (Y6). Fifty-seven schools in and around Bristol, UK, participated in Y1, with 47 and 50 of these participating in Y4 and Y6 respectively, with all children in the relevant year group eligible to take part. The Active-6 study [13, 16, 17, 28] was designed to investigate the impact of COVID-19 on children’s physical activity by comparing the Phase 3 B-Proact1v data on 10-11-year-olds to new data collected after the 2020/21 lockdowns. Data was collected in two waves: Wave 1 in June-December 2021 (Y6-W1) and Wave 2 in January-July 2022 (Y6-W2). Fig 1 shows the timings of the five samples. We invited the same 50 schools from BProact1v Y6 to participate, with 23 and 27 schools agreeing in Y6-W1 and Y6-W2 respectively (22 schools participated in both waves). Both studies collected accelerometer and questionnaire data from children in Year 6 (aged 10–11 years). Both studies received ethical approval from the School of Policy Studies Ethics Committee at the University of Bristol with parental informed consent obtained online or in writing for all participants. Only the Project Manager had access to participants’ identifiable information, which was stored separately from the main dataset.

### Data

In total, data were collected from 1299 children at Y1, 1223 at Y4 and 1296 at Y6 from B-Proact1v, and 393 and 436 children in Y6-W1 and Y6-W2 from Active-6, respectively. Children wore a waist-mounted ActiGraph wGT3X-BT accelerometer (Actigraph LLC; Florida, US) during waking hours for five consecutive days including two weekend days in B-Proact1v, and for seven consecutive days in Active-6. An open-source R script [29] was used to process the accelerometer data. We excluded data between midnight and 6am and defined a valid day as at least 500 minutes of data, excluding intervals of ≥60 minutes of zero counts and up to two minutes of interruptions [30]. All participants who provided at least two valid weekdays and one valid weekend day of data were included. Evenson population-specific cut-points for children [31] were used to derive mean weekday and weekend minutes of MVPA, LPA and sedentary time from 10s epochs. These were converted to proportions of the total wear time spent in MVPA, LPA and sedentary time. We also calculated whether children met the UK physical activity guidelines of a daily average of 60 min per day.

Child gender and the highest education qualification in the household were reported in a parent/carer questionnaire. Children completed a questionnaire about the frequency with which they engaged in different forms of activity outside school hours: sport or exercise club at school, sport or exercise club elsewhere, playing outdoors in their neighbourhood, and playing outdoors at home (0 = ‘Never’, 1 = ‘1–2 days a week’, 2 = ‘3–4 days a week’, 3 = ‘5–7 days a week’). These were coded with the midpoint number of days for each category and averaged to form two variables for the number of days participating in structured (clubs) and unstructured (play) activities, on a scale of 0–6 days.

### Statistical analysis

Descriptive summaries and missing data were reported for all variables. This analysis falls into two parts. Firstly, previous pre-COVID-19 longitudinal results on children’s activity profiles for Y1 and Y4 were extended to Y6. Secondly, the Y6 analysis was repeated cross-sectionally for children post-lockdown (Y6-W1 and Y6-W2) to investigate the potential impact of the COVID-19 lockdowns on activity profiles. For consistency with previous results, we used the same model structure as before [23]. Briefly, the latent profiles were identified based on separate weekday and weekend proportions of time spent in MVPA and sedentary time (with LPA implicit, as proportions sum to one). We assumed a non-zero covariance between MVPA and sedentary proportions, with weekday and weekend variances the same within a profile, but differing for MVPA and sedentary time, and between profiles. All latent variable analyses were performed in MPlus v8.8 [32] and rerun with multiple start values to ensure that the maximum log-likelihood value was replicated. We used full information maximum likelihood, which has been shown to produce unbiased parameter estimates and standard errors under a missing at random assumption [33].

#### Pre-COVID-19 extension to Y6

We fitted cross-sectional latent profile models for 2–10 activity profiles and determined the appropriate number of profiles using a mixture of statistical criteria, interpretability and context (profiles that capture meaningful differences or specific populations of interest) and parsimony (fewer profiles and avoidance of very small profiles preferred), as recommended [34, 35]. Statistical criteria used to measure model fit were the Bayesian Information Criterion (BIC) and the sample-size adjusted BIC (saBIC) where lower values indicate better fit [36]. To compare models with different numbers of profiles, we reported the Lo-Mendell-Rubin (LMR) [37] and bootstrapped likelihood ratio (BLRT) tests [38]. These tests compare a k-1 versus a k-class model, with a low p-value rejecting the k-1 class model in favour of the k class model. We also reported the entropy, which measures the separation between classes, with higher values denoting better separation, although we do not use this to determine the optimum number of classes [39]. Descriptive labels were applied to each profile. For each profile, we also estimated the expected proportion who met the physical activity guidelines, by calculating the probability of achieving at least 60 mins of MVPA, based on the estimated distribution of proportion of time spent in MVPA and the overall average wear time. To examine change in profile membership between Y4 and Y6, we first tested for measurement invariance (that is, whether the same latent profiles were present at both timepoints) and, if there was evidence to support invariance, we estimated a new joint model. We then used a latent transition model [40, 41] for those children with data at both time points (n = 805), fixing the profiles as specified in either the joint or individual cross-sectional models (depending on measurement invariance), and estimated transition probabilities between Y4 and Y6 profiles.

#### Pre-COVID-19 and post-lockdown cross-sectional comparison

As the focus of these analyses was on the potential impact of the COVID-19 pandemic rather than purely descriptive for each wave, we adopted a slightly different approach in the comparison of pre- and post-lockdown data, by fixing the Y6 pre-COVID-19 activity profiles, and prioritising consistency between different time points where justified. This also avoided issues with smaller profiles being lost due to the smaller sample sizes in the two post-lockdown waves. We used multiple group latent class analysis [21], treating each post-lockdown wave (Y6, Y6-W1 and Y6-W2) as a separate group, as previous analysis has shown differences in overall average physical activity [13].

We first fit a model with Y6-W1 and Y6-W2 latent profiles fixed to be the same as the Y6 latent profiles above and compared it to a model that allowed the post-lockdown Y6-W1 and Y6-W2 profiles to differ. A likelihood ratio test was used to test whether the Y6, Y6-W1 and Y6-W2 latent profile definitions were the same (test for measurement invariance). If activity profile definitions differed, we further tested (a) whether Y6 and Y6-W2 were the same (reflecting a hypothesis of an initial change in profiles at Wave 1 followed by a return to previous profiles) and (b) whether Y6-W1 and Y6-W2 were the same (reflecting the hypothesis that pre and post lockdown activity profiles differ). The final model was used to produce estimates of the proportions of children in each activity profile at each time point. We also examined whether there were differences in gender, household education and structured/unstructured activities between profiles with a Wald test via the BCH method, which includes the classification error and is robust to assumption violations [42]. Accelerometer wear time may potentially affect estimates of MVPA/LPA/sedentary proportions and hence could influence profile membership probabilities. To check whether this was an issue we also tested for differences in wear time between profiles.

## Results

Missing data (S1 File: Table 1) were similar across Y6, Y6-W1 and Y6-W2, with child characteristics missing 0–8% of data and accelerometer data missing 6–30%. Most missing data was due to a child having insufficient weekend accelerometer data. The gender balance was similar across all three samples, with a higher percentage of those from households with higher educational qualifications in the post-lockdown Y6-W1 and Y6-W2 samples. The overall proportion of time spent in MVPA, LPA and sedentary time did not differ markedly (Table 1), but the average number of days children reported engaging in structured and unstructured activity was lower post-lockdown by just under half a day for each. The percentage of children meeting UK physical activity guidelines dropped from 40% pre-COVID-19 to 37% in Wave 1 but returned to 41% in Wave 2.

**Table 1:** Proportions of time spent in MVPA, LPA and sedentary time for age 10–11 samples.

	Pre-COVID-19		Post -lockdown			
	Y6		Y6-W1		Y6-W2	
	N	%	N	%	N	%
% female	680	52%	193	49%	224	51%
% degree or higher	636	53%	257	66%	267	62%
% meeting guidelines	367	40%	105	37%	128	41%
	mean	SD	mean	SD	mean	SD
Structured activity (days)	1.9	1.4	1.5	1.1	1.6	1.3
Unstructured activity (days)	3.0	1.6	2.6	1.5	2.5	1.5
**WEEKDAY**						
Proportion of MVPA	0.08	0.03	0.08	0.08	0.08	0.03
Proportion of LPA	0.28	0.05	0.27	0.05	0.27	0.05
Proportion of sedentary time	0.64	0.07	0.66	0.06	0.65	0.06
**WEEKEND**						
Proportion of MVPA	0.08	0.05	0.09	0.04	0.08	0.05
Proportion of LPA	0.28	0.06	0.27	0.06	0.28	0.06
Proportion of sedentary time	0.64	0.09	0.66	0.09	0.64	0.09

MVPA = moderate-to-vigorous physical activity; LPA = light physical activity

Y6: March 2017-May 2018; Y6-W1: June—December 2021 Y6-W2: January–July 2022

### Pre-COVID-19 cross-sectional profiles at Y6

**Fig 2:**
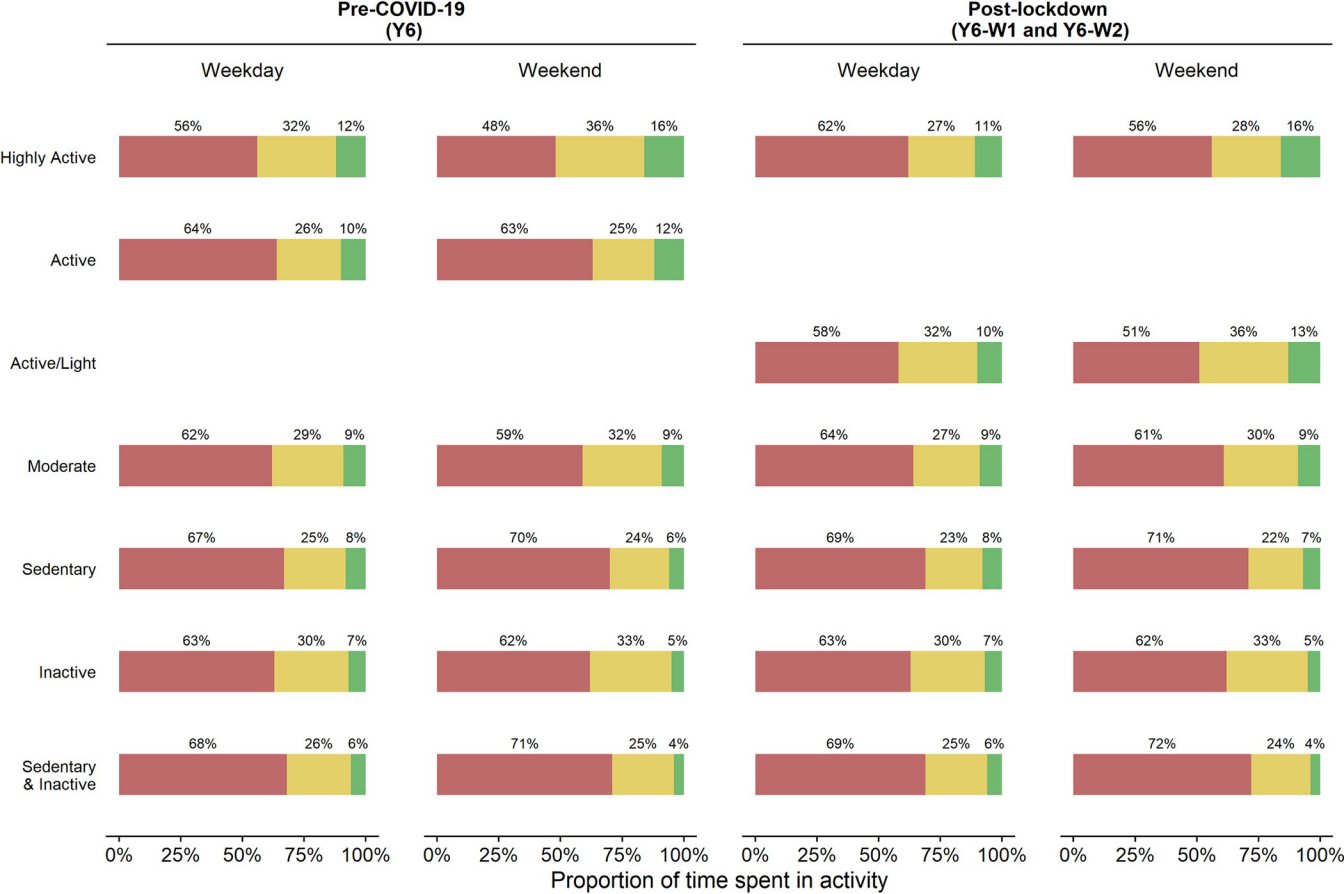
Pre and post-lockdown latent profile definitions for 10-11-year-old children. Red = Sedentary time, yellow-light physical activity, green = moderate to vigorous physical activity.

**Fig 3:**
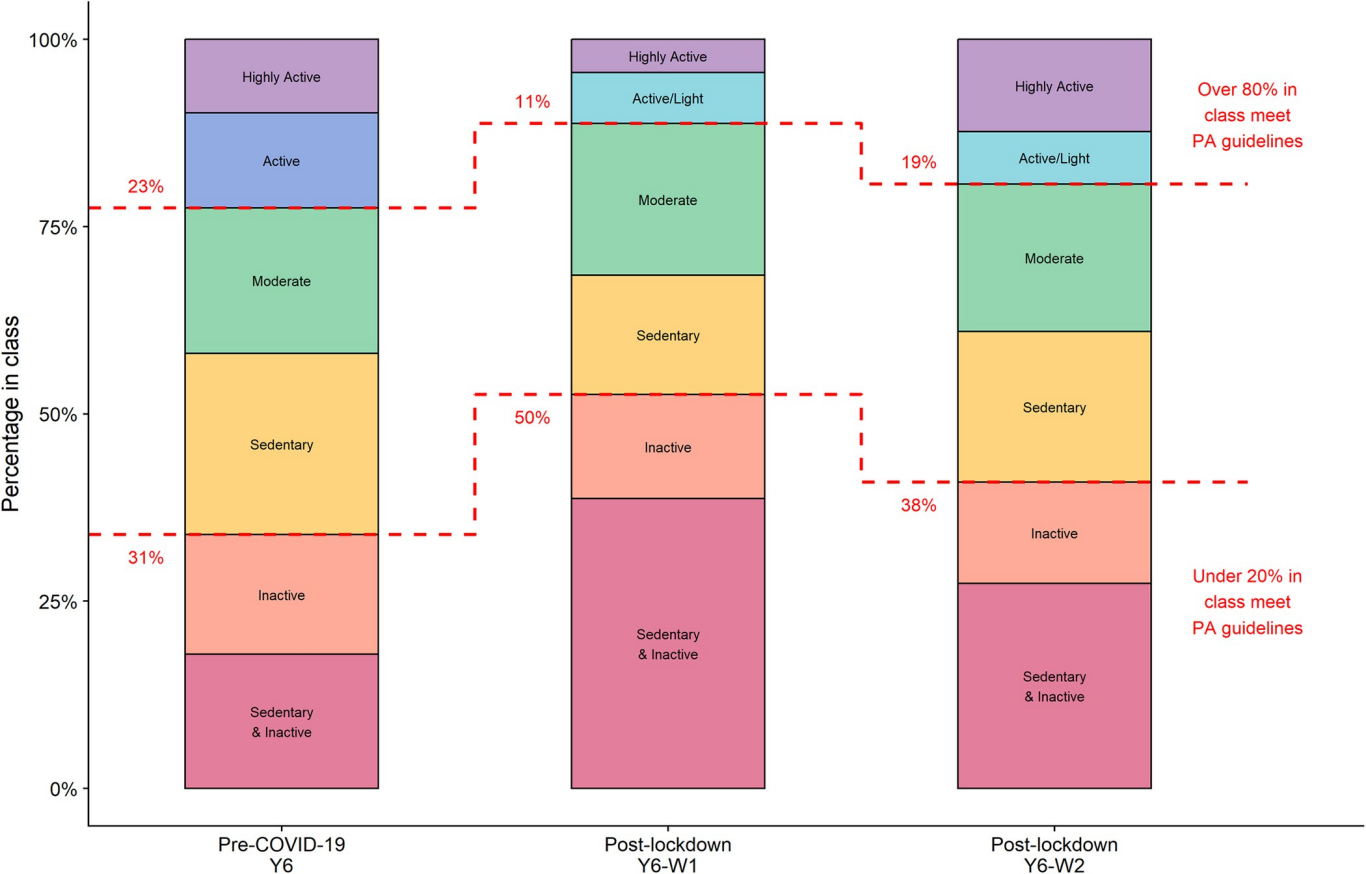
Distribution of Y6, Y6-W1 and Y6-W2 activity profiles pre- and post-lockdown for 10-11-year-old children. Y6: March 2017-May 2018; Y6-W1: June—December 2021 Y6-W2: January–July 2022.

Table 2 gives model fit indicators for the Y6 data for 2–10 profiles. The LMR supported a three-profile model, with BIC and adjusted BIC indicating five or activity profiles, respectively. The five- and six-profile models produced profiles that were similar to those seen in the previously reported Y1 and Y4 analyses. The main difference between five and six profile models was the addition of an extra sedentary and inactive activity profile, comprising 10% of the data. We chose the six-profile model on the basis of interpretability and context, as this additional profile captured a specific population of policy interest, was relatively large and was consistent with the post-COVID-19 lockdown analysis below. The weekday and weekend MVPA/LPA/sedentary proportions for each profile are shown in Fig 2 (left) with the estimated proportion of children in each profile shown in Fig 3 (left) and Table 3; full details of estimates are given in S1 File: Table 2. The six pre-COVID-19 activity profiles, ordered from most active to least (based on estimated proportion meeting physical activity guidelines) were as follows:

- **Highly Active** (10%): High and very high proportions of MVPA, especially on weekends, and low proportions of sedentary time (95% meet guidelines)
- **Active** (13%): Higher than average proportions of MVPA, and typical proportions of sedentary time (83% meet guidelines)
- **Moderate** (20%): Average proportions of MVPA and weekday sedentary time; more LPA at weekends (62% meet guidelines)
- **Sedentary** (23%): Average proportions of MVPA but high levels of sedentary time, with more sedentary time and less MVPA at weekends (28% meet guidelines)
- **Inactive** (15%): Low proportions of MVPA and average levels of sedentary time. (10% meet guidelines)
- **Sedentary & Inactive** (19%): Low to very low proportions of MVPA and high to very high levels of sedentary time (2% meet guidelines)

**Table 2:** Model fit for models with 2–10 classes for Y6.

Number of classes	BIC	saBIC	aLMR p-value	BLRT p-value	entropy	Smallest class
2	-15463.2	-15507.6	<0.001	<0.001	0.629	29%
3	-15622.0	-15688.7	0.015	<0.001	0.599	20%
4	-15684.0	-15773.0	0.139	<0.001	0.568	13%
5	-15696.8	-15808.0	0.536	<0.001	0.564	10%
6	-15691.0	-15824.6	0.158	<0.001	0.547	10%
7	-15675.6	-15831.2	0.666	<0.001	0.576	4%
8	-15657.3	-15835.2	0.202	<0.001	0.573	<1%
9	-15635.1	-15835.3	0.664	<0.001	0.586	<1%
10	-15613.7	-15836.1	No convergence		0.598	<1%

BIC = Bayesian Information Criterion; saBIC = sample-size adjusted BIC; aLMR = adjusted Lo-Mendell-Rubin test; BLRT = bootstrapped likelihood ratio test

^1^ lower BIC indicates better model fit

^2^ p-value for test comparing the current number of classes to a model with one fewer classes

**Table 3:** Percentage of time spent in sedentary (SED), light (LPA) and moderate to vigorous (MVPA) physical activity for pre-COVID-19 and post-lockdown profiles.

		Pre-COVID-19		Post-lockdown	
Class label		Weekday	Weekend	Weekday	Weekend
Highly Active	SED:	56%	48%	62%	56%
	LPA:	32%	36%	27%	28%
	MVPA:	12%	16%	11%	16%
Active	SED:	64%	63%		
	LPA:	26%	25%		
	MVPA:	10%	12%		
Active/Light	SED:			58%	51%
	LPA:			32%	36%
	MVPA:			10%	13%
Moderate	SED:	62%	59%	64%	61%
	LPA:	29%	32%	27%	30%
	MVPA:	9%	9%	9%	9%
Sedentary	SED:	67%	70%	69%	71%
	LPA:	25%	24%	23%	22%
	MVPA:	8%	6%	8%	7%
Inactive	SED:	63%	62%	62%	61%
	LPA:	30%	33%	31%	34%
	MVPA:	7%	5%	7%	5%
Sedentary & Inactive	SED:	68%	71%	69%	73%
	LPA:	26%	25%	24%	23%
	MVPA:	6%	4%	6%	4%

Weekday and weekend patterns were similar within a profile, with weekends typically more extreme versions of weekday patterns. Active profiles (*Highly Active* and *Active*, in which over 80% of children were estimated to meet physical activity guidelines) comprised 23% of the population, while the least active profiles (*Inactive* and *Sedentary & Inactive*, in which under 20% of children were estimated to meet physical activity guidelines) comprised 34%.

### Pre-COVID-19 transition between Y1, Y4 and Y6

**Fig 4:**
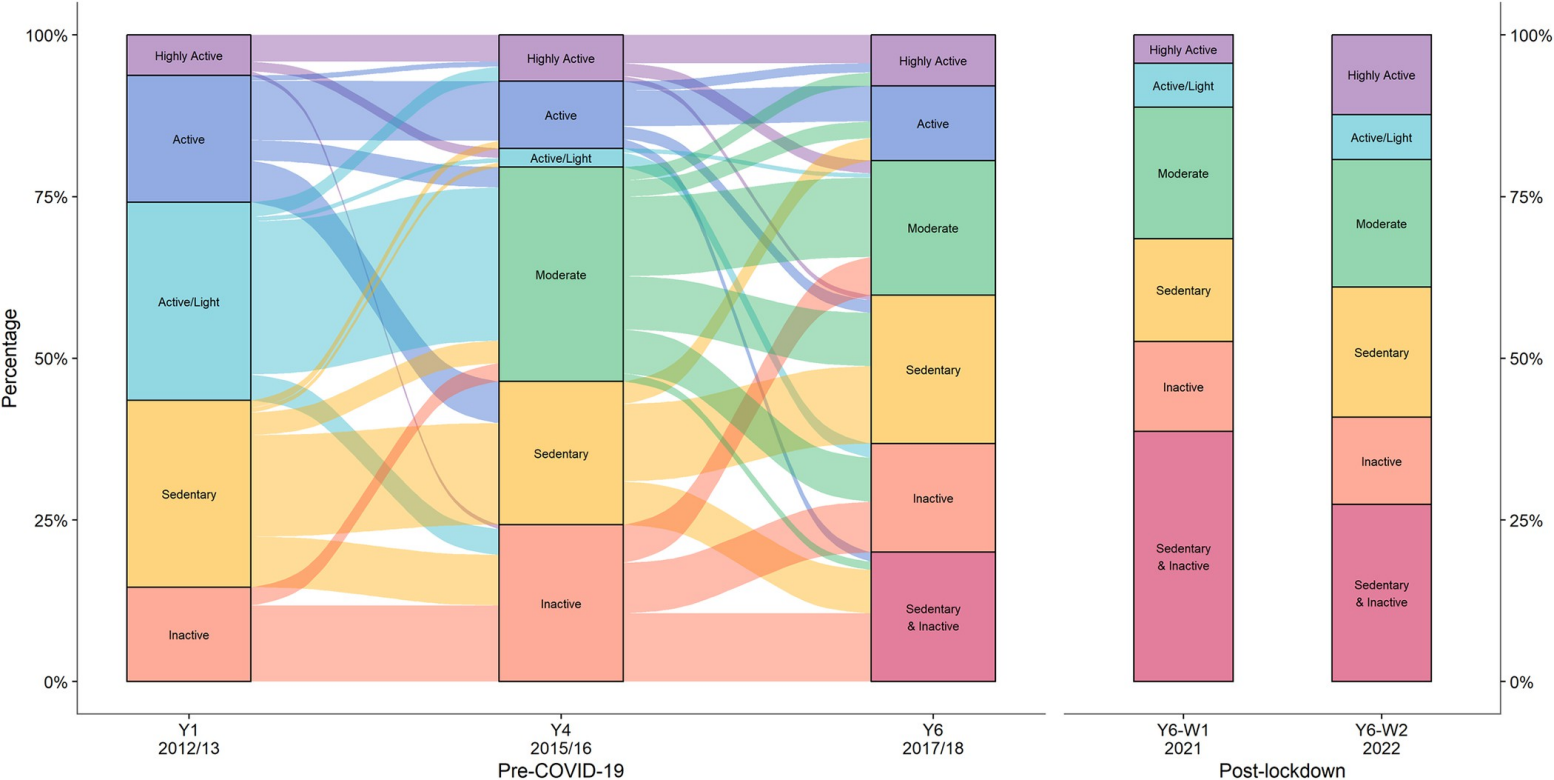
Transitions between activity profiles at Y1, Y4 and Y6, and cross-sectional comparison with Y6-W1 and Y6-W2. Y1: January 2012—July 2013 (age 5–6); Y4: March 2015—July 2016 (age 8–9); Y6: March 2017- May 2018 (age 10–11) Y6-W1: June—December 2021 (age 10–11); Y6-W2: January—July 2022 (age 10–11).

Fig 4 shows the previously reported transitions between profiles at Y1 and Y4 [23] extended to the newly created profiles at Y6, with transition probabilities provided in S1 File: Table 3. Note that activity profile labels from the previous analysis [23] have been relabelled and reordered slightly (see S1 File: Table 4 for further details) so that profiles reported in this paper are ordered from most active (top) to least active (bottom), based on estimated proportion meeting physical activity guidelines.

There was substantial movement between profiles between Y4 and Y6 (Fig 4; S1 File: Table 3). The *Active/Light* profile, characterised by higher-than-average MVPA and high levels of LPA, reduced in size from 29% to 6% between Y1 and Y4, and disappeared completely from the Y6 profiles. The *Sedentary & Inactive* profile, characterised by very low MVPA and very high sedentary time, emerged as a new activity profile at Y6, with those in the *Sedentary* and *Inactive* profiles at Y4 most likely to move to this profile. Overall, the most common patterns of movement were to a profile with a lower MVPA (45%) or no change (36%), with only 19% moving to a more active profile.

### Pre-COVID-19 and post-lockdown cross-sectional comparison

We estimated six latent profiles for Y6, Y6-W1 and Y6-W2 with the pre-COVID-19 Y6 profiles fixed to be those estimated above. Likelihood ratio tests suggested that activity profiles differed between waves, with additional tests concluding that the differences were between pre (Y6) and post (Y6-W1 & Y6-W2) lockdown, with profiles in the post-lockdown waves the same, although with different proportions (Table 4). The post-lockdown activity profiles (Table 3; Figs 2 and 3; S1 File: Table 5) are described below, with the Y6-W1 and Y6-W2 percentages given in brackets, and highlighting similarities and differences to the pre-lockdown profiles:

- **Highly Active** (Y6-W1:5% Y6-W2: 12%): High and very high proportions of MVPA, especially on weekends, and low proportions of sedentary time. Similar MVPA to *Highly Active* pre-COVID-19 profile, but more sedentary. (96% meet guidelines)
- **Active/Light** (Y6-W1: 7% Y6-W2: 7%): higher than average proportions of MVPA, and low proportions of sedentary time, both more extreme on weekends. This profile differs from the pre-COVID-19 *Active* profile, with similar MVPA, but more LPA/less sedentary time. (82% meet guidelines)
- **Moderate** (Y6-W1: 20% Y6-W2: 20%): Average proportions of MVPA and weekday sedentary time; more LPA at weekends. Similar MVPA to *Moderate* pre-COVID-19 profile, but more sedentary, and fewer meet the guidelines. (58% meet guidelines)
- **Sedentary** (Y6-W1: 16% Y6-W2: 20%): Average proportions of MVPA but high levels of sedentary time, and more sedentary at weekends. Slightly more weekend MVPA and weekday sedentary compared to *Sedentary* pre-COVID-19 profile, and more likely to meet the guidelines. (42% meet guidelines)
- **Inactive** (Y6-W1: 14% Y6-W2: 13%): Low proportions of MVPA and average levels of sedentary time. Very similar to *Inactive* pre-COVID-19 profile. (8% meet guidelines)
- **Sedentary & Inactive** (Y6-W1: 38% Y6-W2: 27%): Low to very low proportions of MVPA and high to very high levels of sedentary time. Similar MVPA to *Sedentary & Inactive* pre-COVID-19 profile, but more sedentary. (3% meet guidelines)

**Table 4:** Likelihood ratio tests for pre- and post-lockdown profile comparison.

Model		log-likelihood	df	p-value^1^
1	Different profiles for each wave	11300.2	104	
2	Same profiles across all waves	11261.5	56	0.005
3	Recovery model (Y6 = Y6-W2)	11250.6	67	<0.001
4	Pre/post COVID-19 model (Y6-W1 = Y6-W2)	11279.8	67	0.307

Pre-COVID = 19 Y6 profiles fixed

df = degrees of freedom.

^1^ p-value for likelihood ratio test comparing the specified model to Model 1.

The largest difference between pre- and post-lockdown activity profiles was the replacement of the pre-COVID-19 *Active* profile (characterised by higher MVPA and average sedentary time) with an *Active/Light* profile, which had similar MVPA but less sedentary time and more LPA. This profile was similar to the *Active/Light* profile seen in Y4 (although more sedentary, in common with all Y6 activity profiles). In general, post-lockdown activity profiles were more sedentary than pre-COVID-19, particularly the *Highly Active* and *Moderate* profiles, although the proportions of MVPA remained similar for most. There were also differences in the percentage meeting guidelines, with those in the *Moderate* profile slightly less likely to meet guidelines post-lockdown (59% compared to 62%) but those in the post-lockdown *Sedentary* profile more likely (42% compared to 28%).

Although the activity profiles were the same in Y6-W1 and Y6-W2 post-lockdown, there were substantial differences in the proportions within each profile. In Y6-W1 1, only 5% were in the *Highly Active* profile, whereas over a third (38%) were in the *Sedentary & Inactive* profile. In Wave 2, proportions were closer to pre-pandemic, with the exception of the *Sedentary & Inactive* which was higher (27% compared to 19% pre-pandemic). In Y6-W1, over half of children (52%) were in the least active profiles (less than 20% meet guidelines), decreasing to 40% in Y6-W2, compared to 34% pre-pandemic (S1 File: Table 5).

#### Differences by gender, household education and structured/unstructured activities

The *Inactive* and *Inactive & Sedentary* profiles contained the highest proportion of girls at 85% and 69% respectively pre-COVID-19 (Y6), while post-lockdown in Y6-W2 the highest proportions were for the new *Active/Light* profile (86%), with proportions in the *Inactive* and *Inactive & Sedentary* profiles remaining high at 73% and 74% (Table 5). In contrast, over two-thirds (70%) of the pre-COVID-19 Y6 *Highly Active* profile were boys, rising to 82% post-lockdown in Y6-W2. The new post-lockdown *Active/Light* profile was predominately boys in Y6-W1, similar to the pre-COVID-19 Y6 *Active* profile, but switching to a majority of girls by Wave 2. Pre-COVID-19 Y6 differences by household education were in the less active activity profiles, with a higher proportion of those from households with lower education in the *Inactive* profile, compared to more from higher-education households in the *Sedentary* profile included. These differences disappeared in Y6-W1, but returned in Y6-W2, along with differences in the *Highly Active* and *Moderate* profiles, with the Y6-W2 post-lockdown profiles more likely to be from higher-education households. The new *Active/Light* profile included a higher proportion of children from lower-education households, compared to other post-lockdown activity profiles, and compared to the pre-COVID-19 Y6 *Active* profile. The average number of days engaging in structured activities was highest for the *Highly Active* and *Active* profiles, but lower for *Active/Light* in Y6-W2 with all post-lockdown activity profile averages lower than those in their pre-COVID-19 counterparts (Table 6). Pre-COVID-19 Y6 differences in unstructured activities (highest in *Active* and *Moderate* profiles) were not evident post-lockdown. There were no differences in accelerometer wear time between profiles (S1 File: Table 6), indicating that the distribution of activity profiles was not dependent on wear time.

**Table 5:** Model-based estimates of gender and household education proportions in each activity profile, and test for differences across profiles.

	Female (%)			No Degree (%)		
	Y6	Y6-W1	Y6-W2	Y6	Y6-W1	Y6-W2
All	52%	49%	51%	47%	34%	38%
Highly active	30%	4%	8%	50%	27%	31%
Active	24%			42%		
Active/Light		16%	86%		36%	65%
Moderate	49%	36%	39%	46%	29%	33%
Sedentary	48%	41%	42%	35%	23%	21%
Inactive	85%	59%	73%	63%	43%	84%
Sedentary & Inactive	69%	67%	74%	46%	35%	35%
P-value^1^	<0.001	<0.001	<0.001	0.006	0.637	0.003

^1^ P-value for a Wald test for no difference in covariate between profiles

Y6: March 2017-May 2018; Y6-W1: June—December 2021 Y6-W2: January–July 2022

**Table 6:** Model-based estimates of mean number of days in structured and unstructured activities in each activity profile, and test for differences across profiles.

	Structured Activity			Unstructured Activity		
	Y6	Y6-W1	Y6-W2	Y6	Y6-W1	Y6-W2
All	1.9	1.5	1.6	3.0	2.6	2.5
Highly active	2.7	1.5	2.5	2.9	2.3	2.9
Active	2.4			3.3		
Active/Light		1.9	1.3		3.3	2.5
Moderate	2.1	1.6	2.0	3.3	2.9	2.5
Sedentary	1.7	1.5	1.3	2.7	2.6	2.1
Inactive	1.6	1.5	1.2	3.1	2.3	1.9
Sedentary & Inactive	1.5	1.4	1.4	2.7	2.6	2.6
P-value^1^	<0.001	0.805	0.003	0.012	0.401	0.409

^1^ P-value for a Wald test for no difference in covariate between profiles

Y6: March 2017-May 2018; Y6-W1: June—December 2021 Y6-W2: January–July 2022

## Discussion

This paper has explored pre-COVID-19 longitudinal change in children’s activity profiles between ages 5–6 and 10–11, and compared cross-sectional profiles at ages 10–11 at two time points before and after COVID-19 lockdowns. Pre-COVID-19, we found that activity profiles changed between ages 8–9 and 10–11, with a move away from profiles with high proportions of LPA (*Active/Light*) and the emergence of a new activity pattern characterised by a combination of very low proportions of MVPA and high proportions of sedentary time (*Sedentary & Inactive*), in which only 2% were estimated to meet UK physical activity guidelines. Overall, 45% of children moved to a less active profile at age 11, and a considerable proportion of children became less active and more sedentary, in particular those who were less active to begin with. COVID-19 lockdowns have disrupted children’s activity [13], and we saw substantial differences between pre-COVID-19 in 2018, Y6-W1 in 2021 and Y6-W2 in 2022. These differences could be due to other factors that have changed over this time, rather than a direct consequence of the pandemic, for example, a secular trend, changes to physical activity provision or other events such as increased cost of living, although our results suggest a period of disruption in 2021–22, especially among the most and least active profiles. Post-lockdown, we found: 1) a change in the activity profiles themselves, with an activity profile characterised by high proportions of MVPA (*Active*) replaced by one characterised by a mix of MVPA and light activity (*Active/Light)*, and all activity profiles more sedentary than pre-COVID-19; 2) a change in the proportions of children in each activity profile with a higher proportion of children in the least active profiles, especially in 2021; and 3) changes in the gender and socioeconomic composition of profiles. These all point to important changes in children’s physical activity by 2022, in terms of who is being active and how, that need to be understood before developing new strategies to increase children’s physical activity.

The profiles identified here are similar to those observed in other studies, ranging from very active to inactive [23–25, 43]. In the pre-COVID-19 longitudinal analysis, the increase in sedentary time between Y4 and Y6 and the new Sedentary and Inactive profile, consisting of about a fifth of 10-11-year-olds, reflect a general pattern of lower physical activity and higher sedentary time observed with age [7, 30]. Unstructured activities, such as active play, have also been found to decline with age [23, 44] and may explain the shift away from the Active/Light profile (characterised by unstructured activity) towards structured activity (more common in Highly Active and Active profiles). A third of children stayed in the same profile, while only a fifth moved to a more active profile, similar to the transition patterns seen between Y1 and Y4 [23]. It may therefore be more productive to encourage children to retain physical activity levels at an earlier age, rather than to become active again once they have stopped, especially as children’s physical activity tracks into adulthood [4].

Post-lockdown, the pre-COVID-19 *Active* profile, a more moderate version of the *Highly Active* profile with slightly less MVPA and more sedentary time, was replaced by an *Active/Light* profile, characterised by high levels of LPA, similar to that seen pre-COVID-19 at Y1 and Y4 but which previously disappeared in Y6. Differences in the activity levels and socio-demographic composition suggest that these two profiles capture different activity patterns, reflecting underlying differences in the types of physical activity and who is engaging in them. This difference in physical activity patterns is supported by our qualitative evidence [18] which has found a ‘new normal’ for children’s physical activity in 2022, a year following the COVID-19 lockdowns, with a greater dependence on organised activities. On average, those in the *Active/Light* profile attended fewer active clubs than other activity profiles in which the majority met physical activity guidelines, and so this small group of atypical children may struggle to maintain activity levels in the ‘new normal’. Given the large differences between Y6-W1 and Y6-W2, it is not clear if this profile is a permanent change, or if activity profiles are still changing, and that the *Active/Light* profile may disappear in the future as it has in the past. Pre-COVID-19, children in the *Active/Light* profile in Y4 were most likely to move to the less active *Moderate* (30%) or *Inactive* profiles (39%). Thus, post-lockdown it is important to make sure that children with this activity profile are encouraged to maintain their physical activity. Future research should explore the reasons for their lower participation in structured activities, to ensure accessibility for all and a range of both structured and unstructured active options.

Profiles were generally more sedentary post-lockdown compared to pre-COVID-19, especially in the *Highly Active* profile, where the average proportion of MVPA was similar to pre-COVID-19 but the average proportion of time spent sedentary increased from 56% to 62% on weekdays and 48% to 56% on weekends. While evidence on children’s physical activity after lockdowns is still limited, this is consistent with our previous findings that overall average sedentary time remained lower by 13 minutes on average a year after UK lockdowns were lifted [16], with some lockdown habits, such as increased screen-viewing, continuing [18, 45]. Findings are also conceptually consistent with a Texas-based longitudinal study [15] that found larger increases in sedentary time of 50 min, with only 15% of 8-11-year-olds decreasing their sedentary time between 2019/20 and 2021/22 (although some of this increase may be attributable to age-related rather than COVID-19 changes). Moreover, we found that the proportion of children in the least active profiles (*Inactive* and *Sedentary & Inactive*; those in which fewer than 20% of children were estimated to meet physical activity guidelines) rose from 34% pre-COVID-19 to 52% when lockdowns were first lifted and remained higher than pre-pandemic at 40% a year later. Most notably, the *Sedentary & Inactive* profile doubled in size between pre-COVID-19 and 2021 to 38%. Although smaller by 2022, it remains the largest activity profile, with over a quarter of children (27%), which is concerning given that children in this profile are much less likely to meet physical activity guidelines than those in other profiles. The negative health impacts of sedentary behaviours may be independent of inactivity [2, 46], and so these children are potentially at risk of a double negative health impact. Thus, we urgently need strategies to target this increasingly inactive and sedentary population.

In this study we saw differences in activity patterns by gender and household education, with disparities widening between 2018 and 2022. Pre-COVID-19, the *Highly Active* profile was male-dominated, with only a quarter being girls, but post-lockdown girls were much less likely to have this activity profile, with only 8% female in 2022. So, while overall the proportion of children in the *Highly Active* profile was similar, this was driven mainly by an increase in boys in this group. The opposite pattern occurred in *Sedentary & Inactive* profile, with a higher proportion of girls post-lockdown. The Active Lives Children and Young People Survey 2021–22 reported that the gender gap in physical activity closed during the pandemic, mainly as boys became less active [47], and re-emerged post-lockdown [48] similar to before COVID-19. Our results show the gap is wider for the most and least active profiles than pre-pandemic, and suggest that averaging over all children, as in our previous analysis, may mask the extent of the gender gap in specific subgroups. Similarly, we saw increased differentiation in activity profiles post-lockdown by socioeconomic position, with a lower proportion of children from households with lower education attainment in the *Highly Active* profile, and a higher proportion in the *Inactive* profile. Previous research has suggested that reasons why girls may be less active than boys include lack of role models, desire for more choice and issues around body image [49], and it is possible that these have become more pronounced after lockdowns. Similarly, reasons for socioeconomic gaps include the physical environment, parental education, financial resources and accessibility [50–52]. While the first two factors are unlikely to have changed over the time scale of this study, both cost and accessibility, for example impacted by the COVID-19 pandemic and/or rising cost of living, could potentially explain the widening gaps, especially with the observed stronger reliance on structured activities. Further research is needed to focus specifically on girls and those from lower socio-economic backgrounds to understand why these groups in particular are now less likely to be in the highly active profiles, especially in relation to accessibility and cost of provision, in order to develop interventions that target these groups to prevent these gaps widening still further.

Post-lockdown, the changes in activity profiles suggest that physical activity patterns have become more polarised. While overall averages suggest a recovery in children’s physical activity to pre-pandemic levels [16, 48], this may be masking an important change in the loss of an active ‘middle ground’ with children separating into more or less active groups. This may be further exacerbated if the *Active/Light* profile disappears in future, especially since it consists predominantly of girls (86%) and children from lower socio-economic background (65%), two groups who are typically less likely to meet physical activity guidelines [7]. If, as suggested by our previous qualitative work [18], physical activity is increasingly characterised by structured activities such as active clubs, the danger is that these become the preserve of those who are already active, dominated by boys and those from higher socioeconomic backgrounds, with others becoming more sedentary and inactive. Reduced levels of physical activity among those less likely to be active could thus lead to poorer health and well-being and the widening socio-economic inequalities in health. It is therefore imperative that we ensure active options are available for all abilities and interests rather than just catering to those who are already active.

The key implications of this study are therefore:

- changes in who is being active and how, in particular the increase in the most sedentary profile, may require different strategies in future to increase physical activity;
- those children who currently get their MVPA from less structured sources may need additional support as post-lockdown physical activity becomes more focused around structured activities;
- the widening gender and socio-economic gaps need to be better understood and addressed;
- opportunities to be active need to be accessible to all not just those who are already active.

### Strengths and limitations

Latent class/profile analysis allows us to explore and characterise the complex relationship between physical activity and sedentary time beyond population averages. A key strength of our analysis is that we collected data using the same protocols at all timepoints. This is particularly important as latent profile analysis is a data-driven approach, and so we were able to use the same model approach and structure to estimate the full pre-COVID-19 longitudinal transition between ages 5/6 and ages 10/11, and comparable pre and post-lockdown models. Unlike cluster analysis, a similar approach for identifying groups, latent profile analysis is probabilistic. As a result, our conclusions are more statistically robust as we were able to formally test hypotheses about differences in profiles between waves rather than relying on subjective comparisons. In addition, pre- and post-lockdown samples were recruited from the same schools, reducing the impact of school differences. However, we cannot say that the differences observed between the two time points are directly due to the COVID-19 pandemic or associated lockdowns, as any age-specific activity profiles may change over time naturally, or differences may be due to other factors, for example, changes physical activity provision in both the school and community, changes in school priorities or the increase in cost of living. We were also unable to look at transitions between post-lockdown activity profiles, due to insufficient longitudinal data, or clustering within schools. Children and schools taking part in both B-Proact1v and Active 6 were relatively active, and the Active-6 sample were more likely to come from degree-educated households, and so our analysis may underestimate the proportion of children in the least active profiles, and our findings may not be generalisable to other schools or areas.

## Conclusions

Children’s physical activity patterns have changed after the COVID-19 lockdowns, in terms of who is being active and how. The key changes are activity patterns and proportions which reflect underlying differences in the types of physical activity children engage in, and an increase in the percentage of children in the least active profiles, where under 20% meet UK physical activity guidelines. We also saw a widening of gender and socioeconomic gaps in physical activity, and increased separation between high and low physical activity levels. The key implication of this work is that age and lockdown-related impacts on children’s physical activity are not the same for all but vary according to their physical activity profile, which in turn is influenced by gender and socio-economic position. Thus, a greater understanding of these differences and how to help the different groups of children to be more active is needed to increase both individual and population levels of physical activity.

## Supporting information

S1 Checklist STROBE statement—checklist of items that should be included in reports of observational studies. (DOCX)

S1 File Additional tables. (DOCX)
