# Supplementary material for: Comparison of children’s physical activity profiles before and after COVID-19 lockdowns: A latent profile analysis
Source: PLoS One. 2023 Nov 27;18(11):e0289344. doi: 10.1371/journal.pone.0289344 (PMC10681209; doi:10.1371/journal.pone.0289344)
Supplement: S1 File — (DOCX) [file pone.0289344.s002.docx]

# Comparison of children’s physical activity profiles before and after COVID-19 lockdowns: a latent profile analysis

Ruth Salway, Frank de Vocht, Lydia Emm-Collison^,^ Kate Sansum, Danielle House, Robert Walker, Katie Breheny, Joanna G Williams, William Hollingworth, Russell Jago

**S1 Additional Tables**

**Table 1:** Missing Data

**Table 2:** Y6 - Profile membership proportions and percentage of time spent in sedentary, light and MVPA

**Table 3:** Estimated transition probabilities: probability a child will move from a profile at Y4 to a profile at Y6

**Table 4:** Relabelled classes for Y1 and Y4

**Table 5:** Post-lockdown Y6-W1 & Y6-W2 - Class membership proportions and percentage of time spent in sedentary, light and MVPA

**Table 6:** Model-based estimates of mean accelerometer wear time in each activity profile, and test for differences across profiles

**Table 1: Missing Data**

|  | Pre-COVID-19 | | Post-lockdown | | | |
| --- | --- | --- | --- | --- | --- | --- |
|  | Y6 | | Y6-W1 | | Y6-W2 | |
|  | N  missing | % missing | N  missing | % missing | N  missing | % missing |
| Total |  | N=1296 |  | N=393 |  | N=436 |
| Gender | 0 | 0% | 2 | 1% | 0 | 0% |
| Household education | 105 | 8% | 5 | 1% | 7 | 2% |
| Structured activity | 11 | 1% | 24 | 6% | 27 | 6% |
| Unstructured activity | 21 | 2% | 24 | 6% | 27 | 6% |
| Weekday accel data | 72 | 6% | 25 | 6% | 28 | 6% |
| Weekend accel data | 332 | 26% | 100 | 25% | 119 | 27% |
| % meeting guidelines | 385 | 30% | 106 | 27% | 122 | 28% |

Y6: March 2017-May 2018; Y6-W1: June - December 2021 Y6-W2: January – July 2022

**Table 2: Y6 - Profile membership proportions and percentage of time spent in sedentary, light and MVPA**

| Class label |  | Weekday | Weekend | % meeting guidelines^2^ | % in class^1^ |
| --- | --- | --- | --- | --- | --- |
| Highly Active | SED:  LPA:  MVPA: | 56%  32%  12% | 48%  36%  16% | 95% | 10% |
| Active | SED:  LPA:  MVPA: | 64%  26%  10% | 63%  25%  12% | 83% | 13% |
| Moderate | SED:  LPA:  MVPA: | 62%  29%  9% | 59%  32%  9% | 62% | 20% |
| Sedentary | SED:  LPA:  MVPA: | 67%  25%  8% | 70%  24%  6% | 28% | 23% |
| Inactive | SED:  LPA:  MVPA: | 63%  30%  7% | 62%  33%  5% | 10% | 15% |
| Sedentary & Inactive | SED:  LPA:  MVPA: | 68%  26%  6% | 71%  25%  4% | 2% | 19% |
| Most active  (over 80% meet guidelines) |  |  |  |  | 23% |
| Least active  (under 20% meet guidelines) |  |  |  |  | 34% |

MVPA=moderate-to-vigorous physical activity; LPA=light physical activity; SED=sedentary

^1^ Estimated percentage in each class

^2^ Estimated percentage in class who meet the recommended daily average of 60 minutes or more of MVPA

**Table 3: Estimated transition probabilities: probability a child will move from a profile at Y4 to a profile at Y6**

|  | **Y6** | | | | | |
| --- | --- | --- | --- | --- | --- | --- |
|  | Highly Active | Active | Moderate | Sedentary | Inactive | Sedentary & Inactive |
| **Y4** |  |  |  |  |  |  |
| Highly Active | 0.51 | 0.00 | 0.29 | 0.20 | 0.00 | 0.00 |
| Active | 0.16 | 0.47 | 0.00 | 0.21 | 0.00 | 0.16 |
| Active/Light | 0.14 | 0.02 | 0.30 | 0.14 | 0.39 | 0.00 |
| Moderate | 0.09 | 0.11 | 0.34 | 0.23 | 0.19 | 0.05 |
| Sedentary | 0.00 | 0.16 | 0.00 | 0.54 | 0.00 | 0.30 |
| Inactive | 0.01 | 0.00 | 0.24 | 0.01 | 0.32 | 0.41 |

**Table 4: Relabelled classes for Y1 and Y4**

Previously, the focus was descriptive and so classes were labelled and ordered to reflect the MVPA/sedentary balance. In the current paper, profiles were labelled and ordered to give a stronger focus on MVPA and % meeting guidelines; previous class labels were retrospectively adjusted for consistency, but definitions have not changed.

| **Classes in**  **this paper** | | **Corresponding classes in previous paper ^1^** | |
| --- | --- | --- | --- |
| **Order** | **Label** | **Order** | **Label** |
| 1 | Highly Active | 1 | Highly Active |
| 2 | Active | 3 | Active/sed |
| 3 | Active/Light | 2 | Active/light |
| 4 | Moderate | 4 | Average |
| 5 | Sedentary | 6 | Inactive/sed |
| 6 | Inactive | 5 | Inactive/light |

^1^ Jago R, Salway R, Lawlor DA, et al. Profiles of children’s physical activity and sedentary behaviour between age 6 and 9: a latent profile and transition analysis. *International Journal of Behavioral Nutrition and Physical Activity* 2018;15:103. doi: 10.1186/s12966-018-0735-8

**Table 5: Post-lockdown Y6-W1 & Y6-W2 - Class membership proportions and percentage of time spent in sedentary, light and MVPA**

|  |  |  |  |  | % in class^1^ | |
| --- | --- | --- | --- | --- | --- | --- |
| Class label |  | Weekday | Weekend | % meeting guidelines^2^ | Y6-W1 | Y6-W2 |
| Highly Active | SED:  LPA:  MVPA: | 62%  27%  11% | 56%  28%  16% | 97% | 5% | 12% |
| Active/Light | SED:  LPA:  MVPA: | 58%  32%  10% | 51%  36%  13% | 92% | 7% | 7% |
| Moderate | SED:  LPA:  MVPA: | 64%  27%  9% | 61%  30%  9% | 59% | 20% | 20% |
| Sedentary | SED:  LPA:  MVPA: | 69%  23%  8% | 71%  22%  7% | 42% | 16% | 20% |
| Inactive | SED:  LPA:  MVPA: | 62%  31%  7% | 61%  34%  5% | 11% | 14% | 13% |
| Sedentary & Inactive | SED:  LPA:  MVPA: | 69%  24%  6% | 73%  23%  4% | 2% | 38% | 27% |
| Most active  (over 80% meet guidelines) |  |  |  |  | 12% | 19% |
| Least active  (under 20% meet guidelines) |  |  |  |  | 52% | 40% |

MVPA=moderate-to-vigorous physical activity; LPA=light physical activity; SED= sedentary

^1^ Estimated percentage in each class

^2^ Estimated percentage in class who meet the recommended daily average of 60 minutes or more of MVPA

Y6-W1: June - December 2021 Y6-W2: January – July 2022

**Table 6: Model-based estimates of mean accelerometer wear time in each activity profile, and test for differences across profiles**

|  | Weekday wear time (min) | | | Weekend wear time (min) | | |
| --- | --- | --- | --- | --- | --- | --- |
|  | Y6 | Y6-W1 | Y6-W2 | Y6 | Y6-W1 | Y6-W2 |
| All | 743 | 740 | 738 | 686 | 683 | 696 |
| Highly active | 718 | 738 | 733 | 666 | 681 | 686 |
| Active | 746 |  |  | 687 |  |  |
| Active/Light |  | 736 | 714 |  | 733 | 684 |
| Moderate | 746 | 765 | 754 | 695 | 673 | 708 |
| Sedentary | 734 | 727 | 756 | 681 | 687 | 666 |
| Inactive | 747 | 716 | 734 | 693 | 685 | 686 |
| Sedentary & Inactive | 755 | 742 | 725 | 682 | 680 | 724 |
| P-value^1^ | 0.152 | 0.206 | 0.357 | 0.798 | 0.850 | 0.439 |

^1^ P-value for a Wald test for no difference in wear time between profiles

Y6: March 2017-May 2018; Y6-W1: June - December 2021 Y6-W2: January – July 2022
